# Qualitative analysis of the perceptions of rural communities about the use of awareness‐raising videos in nutrition and health programs in Benin

**DOI:** 10.1002/fsn3.3246

**Published:** 2023-02-01

**Authors:** Fifali Sam Ulrich Bodjrenou, Waliou Amoussa Hounkpatin, Merveille Bada, Simplice Davo Vodouhe, Céline Termote, Mathilde Savy

**Affiliations:** ^1^ Faculty of Agronomic Sciences University of Abomey‐Calavi Cotonou Benin; ^2^ Alliance of Bioversity International and CIAT Cotonou Benin; ^3^ Alliance of Bioversity International and CIAT Nairobi Kenya; ^4^ MoISA, University of Montpellier, Cirad, Ciheam‐IAMM, Inrae, Institut Agro, IRD Montpellier France

**Keywords:** Benin, communication materials, perception, posters, qualitative research, videos

## Abstract

This cross‐sectional study aims at analyzing the perceptions of mothers, community leaders, and nutrition/health care workers (NHCWs) about using videos in nutrition and health programs compared to posters. In total, we recruited 42 mothers, 39 community leaders, and 30 NHCWs from villages and local organizations in two rural districts in South Benin, Bopa, and Houéyogbé. Learning sessions on *Dietary diversity* and *Hygiene and deworming* were organized using posters and videos. Participants' opinions on pros and cons of videos and posters were collected using individual semi‐structured interviews with NHCWs and *focus group* discussions with mothers and community leaders, then analyzed thematically. Results showed that videos were perceived as more adapted to rural communities than posters because they were in local languages, self‐explanatory, appealing, and captivating. Videos also enabled the dissemination of standardized messages. Globally, participants better‐understood messages from videos than from posters, especially when dealing with dynamic processes. However, the speed of video sequences allowed limited time for self‐reflection and assimilation of certain messages. The absence of electricity and lack of equipment to play videos in villages are also major constraints on the use of videos in such settings. While videos are innovant communication tools that should be promoted to improve motivation and compliance in learnings, they should be preferably used as complements to traditional posters for optimized assimilation of messages.

## Graphical abstract

Awareness‐raising videos in local languages were perceived as highly attractive, understandable, and adapted to rural communities for nutrition and health messages dissemination. Videos are not suitable for all nutrition and health messages and their use in rural areas condition faces some constraints such as the absence of electricity and lack of adequate equipment.

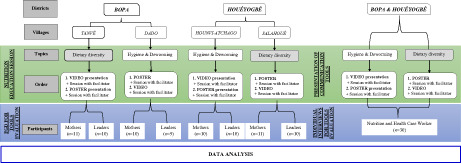

## 1 INTRODUCTION

Nutrition and health sensitization is a strategy for improving the perceptions, knowledge, and behaviors of people regarding nutrition and health practices. It aims at reinforcing awareness and correct unhealthy perceptions and habits, and thus encourage positive behaviors and practices related to food and nutrition. When targeting vulnerable groups in the population, nutrition sensitization programs, either implemented alone or in combination with other types of interventions, may lead to better nutrition outcomes in indicators such as child growth performance (Lassi et al., 2013; Shi & Zhang, 2011; Waswa et al., 2015). Nutrition and health sensitization programs use a wide variety of learning methods, ranging from oral communication to visual aids as communication tools to illustrate, explain, recall or represent ideas, situations, and processes (FAO, 2018; Imdad et al., 2011; Sethi et al., 2003). Other examples of communication media include pictures and drawings, photos, cards, posters, diagrams, gestures, or mimed sequences.

Learning videos are increasingly used and seen as an innovative approach to behavior change in many sectors, particularly health and nutrition (Granger et al., 2015; Morley, 2009). Videos' auditory and visual aspects can be very effective, especially when content is adapted to the contexts and realities of intended audiences (Morley, 2009). Videos create a learning opportunity for any audience but are particularly useful and effective with low‐instruction individuals or populations (Morley, 2009; Sobel et al., 2009; Wlodkowski, 2008). Despite this potential, learning videos are scarcely used in nutrition and health programs in low‐income countries. Considering the case of Benin, a West African country, Bentley et al. (2014) documented one experience with the use of videos in the field of agriculture. They showed that videos facilitated the adoption of rice farming or new agricultural technologies; some farmers also visited extension agencies to get rice seeds and information. The authors highlighted that farmers remembered the content of the videos 5 years afterward. Regarding the nutrition area, the government recommends the promotion of videos during nutrition sensitization sessions at the national level (CAN, 2017). Recently our research team (Bodjrènou, 2021; Bodjrenou et al., 2020) conducted a study to assess the actual effectiveness of learning videos compared to more conventional communication tools – posters and flyers – in improving complementary feeding practices in a food‐insecure area of Southern Benin where these practices were shown to be suboptimal (Bodjrènou et al., 2021; INSAE & PAM, 2017; Mitchodigni, Amoussa Hounkpatin, Ntandou‐Bouzitou, Avohou, et al., 2017; Mitchodigni, Amoussa Hounkpatin, Ntandou‐Bouzitou, Termote, et al., 2017). Results showed that videos did not perform better than traditional tools in improving young children's dietary diversity and meal frequency (Bodjrenou et al., 2020); however, videos were associated with improved knowledge on exclusive breastfeeding as well as with improved practices regarding breastfeeding on demand and increasing breastfeeding frequency during child's illness (Bodjrènou, 2021). In order to better explain these mixed findings, and to investigate the operationalization of learning videos in an African rural context, we did set up a qualitative survey with mothers, community leaders, and nutrition/health care workers (NHCWs) in the same two districts. Our objectives were to (i) assess how widely videos are used as communication tools in nutrition and health sensitization sessions in this area, and (ii) analyze users' perceptions of the videos, particularly perceptions of people working in the fields of nutrition and health at the community level as well as beneficiaries. Our hypothesis is that videos used in nutrition and health programs are perceived by mothers, community leaders, and living in Bopa and Houéyogbé, two rural districts of southern Benin, as more efficient than posters.

## 2 METHODS AND MATERIALS

### 2.1 Study area

This study was conducted in two rural districts located in the department of Mono in Southern Benin, namely Bopa and Houéyogbé, where complementary feeding practices of young children were inadequate (Mitchodigni, Amoussa Hounkpatin, Ntandou‐Bouzitou, Avohou, et al., 2017; Mitchodigni, Amoussa Hounkpatin, Ntandou‐Bouzitou, Termote, et al., 2017) and where a high percentage of households experienced food insecurity (41% in Bopa and 34% in Houéyogbé), as compared to the national rate (10%) (INSAE & PAM, 2017). In 2015–2016, a community nutrition and health sensitization program using videos or posters was implemented in the area for a period of 6 months to improve young children's feeding practices and nutritional status (Bodjrenou et al., 2020). The present study took place in the exact same districts. However, we excluded the villages that had directly benefited from this program in the past, as we wanted to hear from people who had not previously been exposed to these tools. From the list of the remaining villages, we randomly selected two villages per district, namely Dado and Tanvé in the district of Bopa, and Hounvi‐Atchago and Salahoué in the district of Houéyogbé.

### 2.2 Study population and selection of participants

We conducted a cross‐sectional survey combining qualitative and quantitative approaches, which targeted mothers of children under 2 years old, community leaders at the village level and nutrition and health care workers (NHCWs) at the district level. NHCWs were defined as any person or professional working as an educator in an action or intervention for health, food, or nutrition. We first created an inventory of all institutions and structures intervening in the field of nutrition and health in each of the two districts by consulting the administrative registers and maps of the institutions operating at the district level and by discussing with the administrative authorities. Thirty institutions and structures were identified, including social promotion centers, health centers, NGOs, and interventional projects in nutrition. From each structure, one person actively involved in community activities was selected to participate in the study (*n* = 30). In parallel, we used purposive sampling to select participants at the village level. The inclusion criteria for mothers were: (i) having a child under 2 years old, and; (ii) having lived in the village for at least 2 years. A total of 42 mothers were selected with the assistance of village authorities and were deliberately chosen from diverse sociocultural backgrounds (ethnicity, religion, etc.). Community leaders were selected from each village as follows: the village chief, two of the chief's advisors and at least six other influential people or village leaders (when possible, men and women were equally represented). A total of 39 leaders were selected from all four villages.

### 2.3 Data collection

At the district level, we conducted semi‐structured interviews in French with NHCWs, using interview guides that included both closed‐ and open‐ended questions. Interviewees were asked to make an inventory of the tools their institutions used during nutrition and health sensitization sessions. Next, we gathered their opinions on the relevance of videos in learning sessions in the Bopa and Houéyogbé communities. We also simulated learning sessions on two different topics, using either posters or videos, then investigated NHCWs' opinions and perceptions on both tools. The topics and tools used in this test were directly drawn from the nutrition awareness‐raising program conducted in 2015–2016 in the same area (Bodjrenou et al., 2020): (i) Dietary diversity: presentation of food groups and their roles, concept of a balanced diet, importance of dietary diversity, inventory of local foods within each food group, etc.; (ii) Hygiene and deworming: recommended hand washing occasions for mothers and children, importance of washing hands with soap and water, consumption and conservation of drinking water, sanitation of living area, protection of foods against flies and insects, worms and their effect on health, importance of deworming. For each theme, one poster and one video were produced and used to convey identical messages. Posters were mostly graphic with some text in French. We took care to alternate the order of presentation of both themes and both tools across structures.

**FIGURE 1:**
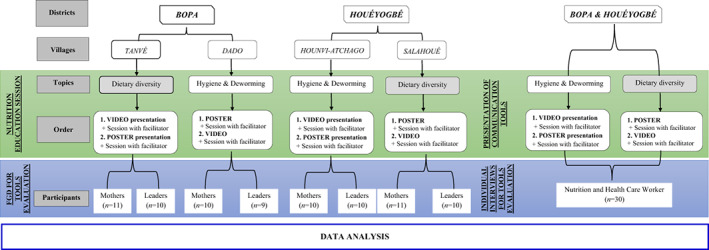
Overall scheme of the study in villages. This figure presents the overall scheme of the study in the two districts. It presents the logic of the investigation including, for each district: the selected villages, the order of presentation of the topics, and the number of participants in *focus group* discussions with mothers in one hand and with community leader on the other hand. FGD, Focus Group Discussion.

In each district, the themes (dietary diversity and hygiene/deworming) were randomly assigned to villages (Figure 1). In each of the four villages, two focus group discussions (FGDs) were held separately using local language, one with mothers and the other with community leaders. Sociodemographic data of participants were collected, including age, gender, ethnicity, religion, level of formal instruction, length of residence in the village, marital status, number of children and income‐generating activities, as well as previous participation to nutrition sensitization sessions and what tools were used during those sessions. As with NHCWs, learning sessions were simulated during FGDs using either posters or videos on the same theme. Video‐based sessions used the following procedure: (i) small groups of four to six participants viewed the video twice on computers; (ii) the facilitator asked participants to share their understanding of the video; (iii) the facilitator paused the video to give additional explanations while soliciting participants' thoughts on the content and use of video as a tool; (iv) participants asked questions and received answers from the facilitator. Poster‐based sessions used the following procedure: (i) the poster was displayed on a wall or board; (ii) the facilitator asked participants to view the poster and explain what they understood; (iii) the facilitator then gave further explanations of the poster's content before opening the floor for discussion.

Furthermore, direct non‐participant observations of participants' attitudes were conducted during all the poster‐based and video‐based sessions. FGDs focused on evaluating the videos in comparison to the posters. Each FGD, which gathered 9–11 participants was facilitated by a moderator, aided by an assistant who was responsible for taking notes on the conduct of the FGD and reminding the moderator of any overlooked elements. Participants provided written consent for photography and audio recording of the discussions. Each FGD used the local language by default, but participants were free to speak in any language, as long as it was understood by all other participants, as checked by the facilitator.

### 2.4 Data processing and analysis

A double entry of quantitative data was carried out and validated using SPSS 22.0 software. Descriptive analyses (frequencies and means) were carried out to determine participants' sociodemographic characteristics and describe nutrition and health NHCWs' feelings about the videos and the messages conveyed therein. Data from the FGDs and interviews were translated from local language into French; data from FGDs and interviews were fully transcribed. Community leaders and mothers' perceptions, opinions, and preferences regarding the videos and posters were manually coded by two researchers and analyzed using a thematic content approach. Discussions occurred in case of coding disagreement. We focused on stakeholders' opinions on the suitability and appeal of the videos (versus posters) to rural populations, as well as the clarity of their intended messages. We evaluated two additional parameters with community leaders and mothers: the ability of videos to hold their attention (concentration) and the ease of memorization of messages. Close direct non‐participant observations of participants' attitudes while watching the videos allowed assessing the concentration. We also used the data for a Strengths, Weaknesses, Opportunities, and Threats (SWOT) analysis of the use of videos and posters in nutrition sensitization programs.

## 3 RESULTS

### 3.1 Sample characteristics

Most of the institutions surveyed in this study have been operating in Bopa and Houéyogbé for more than 10 years (Table 1) and 77% of them had organized nutrition learning sessions. NHCWs were predominantly women. At the village level, the majority of community leaders were over 40 years old, had five children or more and had been living in the community for more than 10 years. All the mothers surveyed were between 15 and 49 years old, and most of them were married and had fewer than five children. The Sahouè was the dominant ethnic group in both mothers' and community leaders' groups. Moreover, 31% of mothers and 59% of leaders had received no formal instruction (Table 2).

**TABLE 1:** Characteristics of nutrition and health care workers and their structures (*n* = 30)

Parameters	*n* (%)
*Sex*	
Male	11 (36.7)
Female	19 (63.3)
*Sectors of intervention*	
Nutrition	29 (96.7)
Health	26 (86.7)
Agriculture	2 (6.7)
Other	6 (20.0)
*Experience in organizing nutrition awareness‐raising sessions*	
Yes	23 (76.7)
No	7 (23.3)
*Duration of working in the districts (workers)*	
<5 years	22 (73.3)
5–10 years	7 (23.3)
>10 years	1 (3.3)
*Duration of operating in the districts (structures)*	
<5 years	2 (7.1)
5–10 years	4 (14.3)
>10 years	22 (78.6)

*Note*: Values presented are numbers, with percentages in parentheses.

**TABLE 2:** Socioeconomic and demographic characteristics of participants of focus group discussions (*n* = 42 for mothers and *n* = 39 for leaders)

Parameters	Mothers	Leaders
*Age*		
15–20 years	11 (26.2)	1 (2.6)
21–29 years	20 (47.6)	5 (12.8)
30–49 years	11 (26.2)	16 (41.0)
50–69 years	0 (0.0)	13 (33.3)
70–80 years	0 (0.0)	4 (10.3)
*Sex*		
Male	–	22 (56.4)
Female	42 (100.0)	17 (43.6)
*Ethnic group*		
Sahouè	28 (66.7)	29 (74.4)
Bopa	7 (16.7)	2 (5.1)
Aïzo	1 (2.4)	6 (15.4)
Others	6 (14.3)	2 (5.1)
*Religion*		
None	7 (16.7)	5 (12.8)
Christianity	26 (61.9)	15 (38.5)
Traditional religion	8 (19.0)	19 (48.7)
Islam	1 (2.4)	0 (0.0)
*Instruction level (years of formal instruction)*		
Illiterate	13 (31.0)	23 (59.0)
Primary school (1–6 years)	19 (45.2)	8 (20.5)
Secondary school, 1st level (7–10 years)	9 (21.4)	6 (15.4)
Secondary school, 2nd level (11–13 years)	1 (2.4)	1 (2.6)
University (14 years or more)	0 (0.0)	1 (2.6)
*Length of residence in the village*		
<5 years	17 (40.5)	3 (7.7)
5–10 years	10 (23.8)	4 (10.3)
>10 years	15 (35.7)	32 (82.1)
*Marital status*		
Married, living with spouse/husband	33 (78.6)	35 (89.7)
Married, living alone	9 (21.4)	2 (5.1)
Widowed/divorced	0 (0.0)	2(5.2)
*Income‐generating activities*		
None	5 (11.9)	0 (0.0)
Agriculture	16 (38.1)	25 (64.1)
Animal husbandry	3 (7.1)	9 (23.1)
Small‐scale trading	20 (47.6)	16 (41)
Trading	1 (2.4)	5 (12.8)
Handicrafts	7 (16.7)	0 (0)
Food processing	3 (7.1)	7 (17.9)
Others	1 (2.4)	9 (23.1)
*Number of children*		
<5	33 (78.6)	11 (28.5)
5–10	9 (21.4)	24 (61.5)
>10	0 (0.0)	4 (10.3)

*Note*: Values presented are numbers, with percentages in parentheses.

We did not observe any differences in the perceptions of participants related to the order of presentation of the two communication media. Nor did participants' socioeconomic and demographic characteristics had any particular influence on their perceptions of the videos and posters. Thus, the presentation of our results does not take these parameters into account.

### 3.2 Inventory of communication media used in nutrition sensitization programs

Among the institutions that organized nutrition awareness‐raising sessions (*n* = 23 out of 30), five (5) did not use any communication media at all. The others (*n* = 18) mainly used, in descending order, image/picture boxes (*n* = 11), posters (*n* = 6), radio broadcasts and theaters (*n* = 2 for both), informational handouts, and cooking demonstrations (*n* = 1 for both); videos were seldom used (*n* = 1). Some institutions used more than one material.

In the villages, 48 out of 81 community leaders and mothers who participated in the FGDs had already benefited from nutritional advice, mainly from health centers. In 29 cases, these nutrition sessions incorporated no communication media. Picture boxes were used in 16 of the cases and posters in 3.

### 3.3 Assessment of learning videos and posters

#### 3.3.1 Appeal and suitability for local communities

Approximately 73% of the NHCWs found that videos were more suited to communicating with local/rural communities than posters. Videos were seen as both an innovative medium and more attractive than posters. An NHCW from Houéyogbé said, ‘People are already accustomed to posters, flyers, picture boxes, etc. Videos are perceived as something new and attractive. Some religions used videos to present their message at village level. And we can easily notice that people appreciate it. Even if they are from another religion, they come and watch videos’. Overall, mothers and community leaders found that the videos were much more engaging and compelling than the posters. Moreover, the effect of the videos extended beyond invited participants, which signifies an opportunity to reach a wider audience. According to a member of the community leaders' group, ‘You probably noticed that as soon as we turned on the video, many other people came to see what was going on as well’. However, both NHCWs and participants at the village level acknowledged that a major constraint related to the use of videos was the lack of electricity and suitable equipment in communities. Some community health centers had televisions, but not regular power, which limited the use of the material despite people's willingness.

#### 3.3.2 Attention and concentration

A significant amount of whispering was observed during poster‐based presentations, as participants were eager to share their understanding of the pictures with each other. This was somewhat disruptive for the facilitator. During video‐based presentations, we observed total calm and attentiveness from the participants, who were totally captivated by the video (Appendix 1). Disruptions nevertheless arose during the sessions because most of mothers came with their children. A mother in Houéyogbé said: ‘For us mothers, it's difficult to stay focused and follow these kinds of sessions because of our children. During the video, my child was crying, so I missed a lot of what was being presented. It's not my fault. But with the poster, even if my child is crying or wants to go to the toilet and I get up, when I come back, the picture is still there and I can always look and ask questions. The video is good, but I think the poster is better for us mothers’. NHCWs were also concerned that participants might be more attracted by the video format itself than by the message. According to them, videos may be too distracting and thus diminish concentration on the messages conveyed, unlike posters.

#### 3.3.3 Clarity of messages/comprehension

**FIGURE 2:**
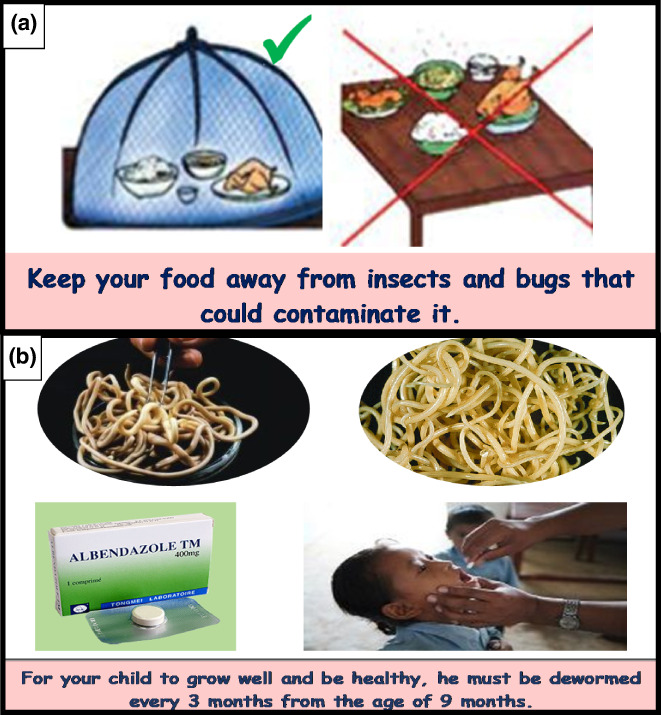
Images from the poster showing the importance of (a) covering food and (b) the prevention of intestinal worms. This figure presents two pictures from posters which had been misunderstood by participants.

Pictures presented in posters were easily misunderstood, and very often the facilitator had to explain the poster several times before participants understood the different messages. Some pictures from the hygiene and deworming poster were considered ambiguous by almost all community leaders and mothers, and participants found them difficult to understand without explanations. For instance, one mother said, ‘I don't understand this picture (Figure 2a). Why using a net to cover meal? We are not used to putting our meals under a net here in our village, but rather we ourselves sleep under the net’. The video, however, showed a woman covering her food, so participants understood the purpose immediately. Likewise, there was some confusion around the photos showing worms (Figure 2b), as most FGD participants in both districts reported seeing ‘something in the picture that looks like a dish of macaroni/spaghetti’. A community leader from Houéyogbé explained, ‘Videos show things better than the poster. For example, in the video on the topic of deworming, we can clearly see how the worms enter the body from dirty hands. On the poster, we don't see that. The facilitator was obliged to explain it to us. With the video, it's really clearer and easier to understand’. The same was true for the video on dietary diversity. ‘The way the roles of different food groups were explained in the video is engaging and facilitates understanding. In the poster, we just saw several photos of foods. We could not know what these mean’, said one mother. However, several mothers highlighted the exhaustive nature of the posters' content as compared to videos; a mother from Houéyogbé commented, ‘The poster related to food diversity shows a greater variety of foods available here in our village as opposed to the video, which shows only some of them. Thus, I prefer the poster to the video’.

Opinions of NHCWs contrasted more than those of respondents at the village level. Approximately half of the former group thought the videos addressed the topic better than the posters, while the reverse was true for 20% of the respondents. For 33% of respondents, there was no difference between the videos and the posters. Most of those who preferred videos to posters pointed out that the videos were in local languages and thus participants could understand their messages without any other explanations, while the posters required further explanations from the facilitator. Globally, topics were better understood through videos than posters, especially when dynamic processes were addressed. After the presentation of videos, participants asked fewer questions related to misunderstandings in comparison to poster‐based presentations; according to interviewees, this was because the videos were clearer than the posters.

#### 3.3.4 Memorization/remembrance

Both mothers and community leaders found the posters to be more effective than videos for medium‐ and long‐term memorization of messages. Static images on the posters were more likely to facilitate memorization, particularly in light of distractions/disruptions that occurred during video presentations. A community leader said, ‘The video is interesting, but I find it very difficult to remember things. So I prefer the poster because the pictures are there in front of me and I can look at the same thing as much as I want. With video, pictures pass quickly and I can quickly forget what I saw’. Some mothers even suggested displaying the posters permanently in a public place, so that everyone from the village could view them as long as they desired.

### 3.4 SWOT analysis

Videos offer major opportunities, including their attractiveness and the clarity of their messages (Table 3). Since the videos were in local languages, they could be self‐utilized, even by people with little or no formal instruction. As videos do not require a facilitator's presence except for during a question‐and‐answer session after the projection, they represent a major advantage in terms of standardization of messages. On the other hand, videos offer limited time for imagination, reflection, and learning processes because of their temporality; they may therefore not be suitable for learning large amounts of messages. Video also requires non‐negligible logistical features, including electricity and specific equipment; however, in settings where these amenities are not a problem, it can be used in both small and large groups. Posters offer the possibility to deliver a large amount of learning messages, but the clarity of messages and learning processes from participants very much rely on the facilitator's skills (speaking skills, interaction with participants, etc.) and on standardization across facilitators. Static images from posters are likely to facilitate memorization and can be displayed permanently so that participants can access the material at will. Since posters are more traditional and common than videos, they may be perceived as an overused medium and thus be associated with a lack of motivation and mobilization from the community.

**TABLE 3:** SWOT (strengths, weaknesses, opportunities, and threats) analysis of the use of videos and posters in nutrition sensitization in selected villages of the districts of Bopa and Houéyogbé.

Strengths		Weaknesses	
Video	Poster	Video	Poster
Attractiveness Standardization and clarity of messages Production in local languages Can be used in large or small groups Adapted to self‐utilization	Static images for step‐by‐step learning, imagination, and memorization Possibility to convey a large amount of information	Limited time imagination and learning (speed of images)	Requires a facilitator Non‐standardization of messages (possible distortion by facilitator) Poor understanding of the images

Opportunities		Threats	
Video	Poster	Video	Poster
Adapted to people with little/no instruction Strong interest and mobilization of the community	Ease of use in villages with low logistics Large‐scale and long‐term use Can be displayed permanently for long‐term use	Distracting format/low attention to content Unavailability of devices to play the videos	Low motivation and mobilization of targets (boring character)

## 4 DISCUSSION

This study which aimed at assessing the place of videos among the communication tools used in nutrition and health awareness‐raising programs showed that videos were seldom used in the districts of Bopa and Houéyogbé, Southern Benin. The National Food and Nutrition Council (CAN, 2017) also reported that health and nutrition institutions from the 77 communes of Benin mainly used picture boxes and posters, while only a handful of health centers had adopted videos. The widespread use of materials based on static pictures, rather than moving pictures, is likely a response to financial and logistical constraints. Video production is technically complex and expensive, and video diffusion requires electricity and specific materials. Most health centers in our study area do not have such equipment, making the use of videos unfeasible in similar contexts without additional specific funding.

Irrespective of the logistics, the use of videos in nutrition programs was perceived relevant to rural communities since they are visual, auditory, attractive, and in local languages, as reported also by Morley (2009). Moreover, videos ‘speak for themselves’, and thus do not necessarily require that someone be present to explain (Bentley et al., 2014). Very often, facilitators are trained people from the community, but their training varies in thoroughness depending on the program's time and budget. Moreover, facilitators' public speaking abilities and pedagogical skills could also be determinant. Thus, the opportunity to present standardized messages through videos seems to be key, particularly in contexts where both the facilitators and the audience are likely to have little or no formal instruction (Bentley et al., 2014; Gagliano, 1988). However, video sessions also demand more self‐discipline from participants (Kay, 2012; Martinez, 2001; Sun & Rueda, 2012). In our study, mother faced many distractions during nutrition sessions and these are mostly linked to children. To address this problem, videos could be made available on demand so that women can watch them as many times as they want, can, and need to. Mobile phones could be a mean to access videos, as their use is now fairly widespread, even in rural communities (Chib et al., 2008; Khan et al., 2018). However, in our study area, smartphones and mobile phones capable of playing videos are usually owned by men and young males, not women; access to videos would therefore typically be at the household level. Alternatively, providing smartphones to community leaders, especially leaders of women's associations, could be another way to organize video‐based awareness‐raising sessions at the community level. This approach would allow exchanges and interactions with peers and offer opportunities for mothers to ask for further explanations after viewing the videos. Making learning videos widely available, that is, to all community members who have suitable devices, can create an environment conducive to behavior change for mothers (Glanz et al., 2008).

Despite their great potential, learning videos still need improvement since some messages could be misunderstood, due to the speed of images for example. In a review, Tuong et al. (2014) reported that videos as communication media can be effective in influencing some health behaviors and not effective for others. In addition, videos content needs to be adapted to rural realities in order to allow participants to identify with the characters in the scenes and conceptualize the information (Ramsay et al., 2012). Videos can be used to illustrate real practices and to visually highlight information that would be impossible to describe orally, in writing or with simple picture. On the other hand, when citing elements/components of the same set (e.g., foods from the same group), posters received a better appraisal. According to Régimbeau (1997) and Mayer et al. (2005), unlike moving pictures that impose their rhythm on the viewer, static images adapt to an individual's ‘time’ and allow him/her to learn at his/her own pace, hence favoring memorization. Therefore, the themes to be addressed during the nutrition sensitization sessions may determine the pertinence of videos or posters as the most appropriate communication medium.

Participants' enthusiasm for video can be great. Even if this is an opportunity for learning, this enthusiasm could be linked to the novelty of videos, and we cannot assume that this phenomenon will persist. Consequently, the use of videos should not be considered a panacea in awareness raising, and a mix of different media may be preferred. Videos are sometimes criticized for their distracting nature, which could obscure the messages to some extent. As shown in a study in India (Wadhwa & Sabharwal, 2018), people enjoyed the nutrition advice provided by videos, but few actually adopted the appropriate behaviors. In the same context in Benin, our research team (Bodjrenou et al., 2020) showed that learning videos were not more effective than posters in improving mothers' complementary feeding practices. Even if the attractiveness of videos is a major advantage, more needs to be done to enable participants to learn something beyond the entertainment aspect. The pedagogy of the educators remains a key element to support individuals in their learning.

## 5 CONCLUSION AND IMPLICATIONS FOR RESEARCH AND PRACTICE

The choice of communication medium should depend on the objectives pursued, the context, and the financial and technical means available. It is important to work upstream of each nutrition sensitization program in order to choose the most appropriate tool for each theme to address. The combination of several communication strategies and of several communication tools should be favored for optimizing learning processes and contributing to the success of awareness‐raising programs. The use of learning videos can be particularly valuable in rural contexts with low instruction. The use of small devices such as smartphones, at individual or small group levels, is suggested to counterbalance the lack of regular electricity and of appropriate equipment, and to promote their self‐utilization.

## 7 FUNDING INFORMATION

This study was funded by the Ministry of Foreign Affairs of Finland and the Agriculture for Nutrition and Health CGIAR Research Program. The first author had also received a PhD allowance from IRD.

8

## 9 ETHICAL APPROVAL

The authors declare that they do not have any conflict of interest. This work has received the ethical clearance of the Benin National Ethics Committee for Health Research (N°45/MS/DC/SGM/DFR/CNERS/SACNERS). In addition, written informed consent was obtained from all study participants.

## Data Availability

The data that support the findings of this study are available on request from the corresponding author. The data are not publicly available due to privacy or ethical restrictions.

## APPENDIX 1 Description of a session in a village

### 1.1 VILLAGE: DADO (BOPA). DATE: JANUARY 22, 2019. FGD WITH MOTHERS

The investigation team, consisting of a moderator and a rapporteur, is welcomed by the village chief while two councilors hurry to gather the participants. Over the next few minutes, almost all of the mothers arrive with their babies on their backs or in their arms.

At 5:24 pm, the ten (10) mothers selected for the study are present under a shed in the village chief's courtyard, and the session can begin.

After the members of the team introduce themselves, they explain to the participants the reason for their presence, the different stages of the session, the instructions and rules of courtesy to be respected and the request for their consent to record the exchanges and take photos. Then, the moderator administers a characterization questionnaire to each mother, while the rapporteur sets up the logistics.

At 5:57 pm, the session starts. The poster on the theme ‘dietary diversity’ is shown to mothers. We observe some participants whispering among themselves; others seem to have come out of obligation and are therefore in a hurry to leave. They are asked to carefully observe and report what they see and how they understand it. Initially, communication is very strained. The moderator suggests the mothers teach him a popular village song. After a few shy laughs, they have a little discussion, and one of them starts a chorus that the others quickly join. The moderator tries to sing and dance with the mothers. After this, the atmosphere is much more relaxed; the mothers start speaking and explain what they understand from the poster. The moderator finally presents the poster, picture by picture. Mothers talked a lot among themselves about their understanding of the poster. After the moderator's presentation, the mothers ask him questions and he provides them with answers.

Next, the video on the same theme is shown. During the film we observe a noticeably high level of attention among the mothers, which was not the case during the exchanges regarding the poster — they are very captivated by the video. Unfortunately, at that moment, it seems some children (under 2 years old) are a little jealous of the attention the video is receiving. We witness the cries and demands of children, which distract the mothers. Some reprimand their children, asking them to be quiet. Unfortunately, some mothers are obliged to attend to their children, thus drawing their focus elsewhere; still, they try to watch the video from a distance.

Moreover, the video attracts the curiosity of the neighbors, who invited themselves early on to observe the session from around the shed. Some women whisper their displeasure at not having been selected to participate in the session. At the end of the video, the mothers ask for a replay. After the replay, the moderator asks the participants what they learned from the video and what they understood. Many concepts are retained, yet somewhat misunderstood or misinterpreted. The moderator then gives further explanations, which are followed by discussions with the mothers.

The nutrition sensitization session ends at 6:46 pm. With the help of a data collection guide, the moderator speaks with participants about their opinions on the poster and video presented, as well as their preference between the two communication media. They give many suggestions and ways to better adapt the content of the poster and the videos to their community. They also express a desire for the tools to be disseminated throughout their community as soon as possible. Another song is sung to close the session. The mothers thank the team for the valuable information and advice they provided and leave happy. Finally, the team puts away their materials and thanks the village chief and his councilors for their collaboration.

At 7:38 pm, the team says goodbye and leaves the village, very satisfied with the session.
